# Integrated Disease Chronicity Modeling to Map Longitudinal Biomarker Relationships in Alzheimer’s Disease

**DOI:** 10.1002/alz.090730

**Published:** 2025-01-09

**Authors:** Seth Robey, Ka Lai Yee, Julie A Stone

**Affiliations:** ^1^ Merck & Co., Inc., Rahway, NJ USA

## Abstract

**Background:**

A lesson of the recent progress in Alzheimer’s Disease therapy is that biomarker‐driven trials will be crucial to demonstrating efficacy in the clinic. Many studies have demonstrated the potential predictive power of fluid and imaging biomarkers in guiding patient selection^1,2^ and continued progress of precision medicine approaches will demand development of multi‐dimensional biomarker arrays. However, correlations between candidate biomarkers change non‐linearly with time, requiring methodologies to align biomarkers across a common disease timescale (time from amyloid positivity; TFAP). We present a disease chronicity analysis using latent time joint mixed effects modeling^3^ (LTJM) which enables prediction of progression from cross‐sectional data.

**Method:**

A model of amyloid PET, tau PET, ADAS‐COG13, MMSE, and CDRSB was estimated with training data from amyloid positive subjects in the ADNI database (N=831). Longitudinal profiles for each endpoint were modeled with a 4‐parameter logistic curve. TFAP was estimated for each subject informed jointly by all biomarkers/endpoints. An external validation set consisted of a random sampling of 76 subjects with longitudinal tau PET and cognitive assessments collected within 3 months prior to the first PET scan (‘baseline data’). Longitudinal predictive power was assessed by projecting disease trajectories from subjects in the validation set, given only the observed baseline data and comparing with the observed post‐baseline data.

**Result:**

Without prior guidance, the model correctly estimated the sequencing of clinical diagnoses (CN, MCI, AD) and the timing of tau and clinical progression are consistent with previous empirical studies; acceleration of clinical progression is closely associated with the onset of tau positivity. When applied to baseline data from the validation set, the model accurately captured the data 2‐7 years post‐baseline across biomarkers, covering the span of a typical trial.

**Conclusion:**

LTJM enables bridging across biomarkers to extract a full progression profile aligned to a common time scale. The model provides unbiased predictions of progression from cross‐sectional data, highlighting its utility for guiding clinical trial patient selection and its potential for refining subject‐level prognoses. Further modeling efforts will incorporate emerging fluid biomarkers and imaging modalities.

Ref: 1) Mattsson‐Carlgren, JAMA Neurol. 2023 Dec; and 2) 2023 Apr; 3) Li, Alzheimers Dement. 2018 Aug

